# On Excision of the Os Calcis, in Incurable Diseases of That Bone, as a Substitute for Amputation of the Foot

**Published:** 1850-07

**Authors:** W. B. Page

**Affiliations:** Surgeon to the Cumberland Infirmary


### SURGERY.

*On Excision of the Os Calcis, in incurable diseases of that bone, as a substitute for amputation of the foot.* By W. B. Page, Esq., Surgeon to the Cumberland Infirmary.

The patient whose case is described in this paper was an unhealthy, ill-nourished, scrofulous boy, aged 16, with disease of the right tarsus, the result of a slight injury he had received several years before. In the beginning of the year 1848, suppuration and ulceration took place; in July, the whole back part of the foot was considerably enlarged, and immediately below the inner ankle was an ulcer, from which several sinuous passages proceeded to the bone, through which a probe could be readily passed into two distinct parts of the substance of the carious os calcis. The disease appeared to be altogether confined to that bone; his health was improved by nourishing diet and cod-liver oil; and in October, further examination, confirming the original impression, that the disease was confined to the os calcis, it was deemed desirable that that bone alone should be removed. The patient being put under the influence of chloroform, Mr. Page made an incision down to the bone in its whole extent, from the lower margin of the ulcer, about half an inch below the inner ankle, directly below the sole of the foot to just below the fibula, which would have enabled him to remove the foot at the ankle-joint if the disease of the tarsal bones had proved more general than he had anticipated. The posterior flap was carefully reflected from the surface of the bone; the insertion of the tendo-Achillis separated, and the joint between the astragalus and calcaneum reached. By the introduction of a small narrow-bladed scalpel he succeeded in dividing the ligamentous structures on either side, and also the inter-osseous ligament. He then made two incisions one on either side of the foot, commencing at the junction of the os calcis with the os cuboides, and ending at the extremities of the first or transverse incision, dissected this flap from the under surface of the bone, readily separated the connections of the calcaneum with the cuboid bone, and after a few touches of the scalpel, the former, with scarcely a particle of soft parts attached, was removed. The astragalus and cuboid bones appeared quite healthy.. For two or three days he went on quite well, but after that suffered much from acute inflammation of the tarsal joints, which resulted in the formation of an abscess in the dorsal surface of the foot. This was opened, the wound speedily healed, and no recurrence of a like nature took place. Erysipelas and phlebitis being very prevalent at that period in the hospital, this patient did not escape, but in him the disease was confined to the affected limb. In January, 1849, he left the hospital, fourteen weeks after the operation, when he was able to bear considerable pressure on the foot without suffering, but he was forbad to wear a shoe or use his foot for some months. Sixteen months after the operation the foot continued sound; when sitting he is able to extend the foot perfectly. The author conclu es with some observations on the desirability of performing this operation in such cases in preference to amputation of the foot.—*Dublin Medical Press.*

